# Wind Resistance Mechanism of an Anole Lizard-Inspired Climbing Robot

**DOI:** 10.3390/s22207826

**Published:** 2022-10-14

**Authors:** Rui Li, Shenyao Feng, Shuang Yan, Xiao Liu, Ping-An Yang, Xingyi Yang, Mengjie Shou, Zhangguo Yu

**Affiliations:** 1 School of Automation, Chongqing University of Posts and Telecommunications, Chongqing 400065, China; 2 Intelligent Robotics Institute, School of Mechatronical Engineering, Beijing Institute of Technology, Beijing 100081, China; 3 Chongqing Menlo Robot Technology Co., Ltd., Chongqing 400799, China

**Keywords:** climbing robot, anole lizard, wind resistance mechanism, bio-inspired robot

## Abstract

The stable operation of climbing robots exposed to high winds is of great significance for the health-monitoring of structures. This study proposes an anole lizard-like climbing robot inspired by its superior wind resistance. First, the stability mechanism of the anole lizard body in adhesion and desorption is investigated by developing adhesion and desorption models, respectively. Then, the hypothesis that the anole lizard improves its adhesion and stability performance through abdominal adjustment and trunk swing is tested by developing a simplified body model and kinematic model. After that, the structures of the toe, limb, and multi-stage flexible torso of the anole lizard-like climbing robot are designed. Subsequently, the aerodynamic behavior of the proposed robot under high-speed airflow are investigated using finite element analysis. The results show that when there is no obstacle, the climbing robot generates the normal force to enhance toepad friction and adhesion by tuning the abdomen’s shape to create an air pressure difference between the back and abdomen. When there is an obstacle, a component force is obtained through periodic oscillation of the spine and tail to resist the frontal winds resulting from the vortex paths generated by the airflow behind the obstacle. These results confirm that the proposed hypothesis is correct. Finally, the adhesion and wind resistance performance of the anole lizard-like climbing robot is tested through the developed experimental platform. It is found that the adhesion force is equal to 50 N when the pre-pressure is 20 N. Further, it is shown that the normal pressure of the proposed robot can reach 76.6% of its weight in a high wind of 14 m/s.

## 1. Introduction

Climbing robots are crucial for health-monitoring and safety maintenance of structures [1,2]. Climbing robots can carry various instruments and equipment to perform inspection and maintenance operations on the surfaces of structures and small interior spaces [3,4,5], such as for high-altitude curtain wall cleaning [6,7,8], ship cleaning [9,10,11], and crack detection [12,13,14].

Most existing studies focus on improving the ability of climbing robots to form stable contact with the surface. For example, La et al. [15] embedded permanent magnets into the wheels to produce adhesion, and each wheel could generate a magnetic force of about 140 N. Nevertheless, the magnetic adsorption mechanism was bulky and consumed much energy for desorption. Li et al. [16] developed a crawler climbing robot based on negative pressure adsorption that could climb walls with a load of 7.26 kg. Similarly, Zhou and Li [17] installed a suction device on the climbing robot’s abdomen to enable it to move on rough surfaces. Liu et al. [18] proposed a novel under-actuated soft adhesion actuator whose good adaptability to various surfaces makes it suitable for a wide range of applications. In addition to the above methods, bionic design has been the main research direction in recent years [19,20]. Murphy and Sitti [21] designed a wall-climbing robot by curing new adhesion materials with a gecko bristle array structure, which had a good climbing performance on flat surfaces. Kim et al. [22] developed the Spinybot series of claw-thorn-type climbing robots inspired by the excellent climbing ability of cockroaches, which can climb on different rough surfaces. Boston Dynamics has successively developed a bionic claw-thorn-type robot that can quickly climb on circular walls and stably climb rough vertical walls [23].

The climbing robots mentioned above showed good climbing performance on different surfaces, but none of them have been designed taking into consideration the effects of high wind on the stability of the robots. When subjected to high wind, climbing robots inevitably generate a normal overturning force that makes it difficult to maintain stability, resulting in deteriorated climbing performance [24,25,26]. Thus, improving the wind resistance of climbing robots is key to enabling them to work stably in high winds.

Nishi and Miyagi [27] designed a climbing robot with a propeller and adopted a joint control system consisting of a thrust controller, a friction intensifier, and a damper. Simulation results proved that the robot could generally work in high winds. Myeong et al. [28] developed a drone-style climbing aerial robot platform that resisted wind disturbances through attitude changes and perching mechanisms to reduce the risk of the robot accidentally falling in high wind. Qian et al. [29] designed a tether-supported wall-sliding robot with sliding suction cups, which employ two closed-loop control modules to regulate the sliding suction cups’ negative pressure to enable them to move reliably in high wind. Xu et al. [30] proposed a new suction method based on the hook-claw mechanism, which can effectively enhance the grasping force to resist the interference of high wind and vibration of buildings. The above research focused on improving the gripping performance and control system to withstand high wind disturbance. Thus, a high driving force and control system are required.

Donihue et al. [31] found that the anole lizards surviving after hurricanes in the Turks and Caicos Islands in the Caribbean Sea have significantly different physical and structural characteristics from common anole lizards. Further, it was found that the self-protection strategy of anole lizards in high winds was not to escape from the perch immediately, but to move quickly behind the perch and then keep the whole body in a soft state by tightening the front limbs, releasing the hind feet, and stretching out the hind limbs. The anole lizards’ unusual physical characteristics and motion behaviors may be the critical factors for their survival in hurricanes. Furthermore, this wind resistance mechanism may provide a new solution for improving the stability of climbing robots.

Therefore, to improve the stability of climbing, this study focuses on designing an anole lizard-like climbing robot inspired by its superior ability to withstand high winds. First, the adhesion and desorption models are developed to investigate the stability mechanism of the anole lizard in the process of adhesion and desorption. Then, based on the developed simplified body model and kinematic model, the hypothesis that the anole lizard improves its adhesion and stability performance in high winds through abdominal adjustment and torso swing is proposed. After that, the structures of the toe, limb, and multi-level flexible torso of the anole lizard-like climbing robot are designed. Subsequently, finite element analysis of the aerodynamic behaviors of the climbing robot in high-speed airflow is conducted using the *k*-ϵ turbulence and shear stress transport models, and the simulation results are employed to verify the proposed hypothesis. Finally, the adhesion and wind resistance performance of the anole lizard-like climbing robot is tested by setting up experimental platforms.

## 2. Stable Climbing Mechanisms of Anole Lizards

### 2.1. Modeling the Adhesion Mechanics of Anole Lizard Toes

**Figure 1:**
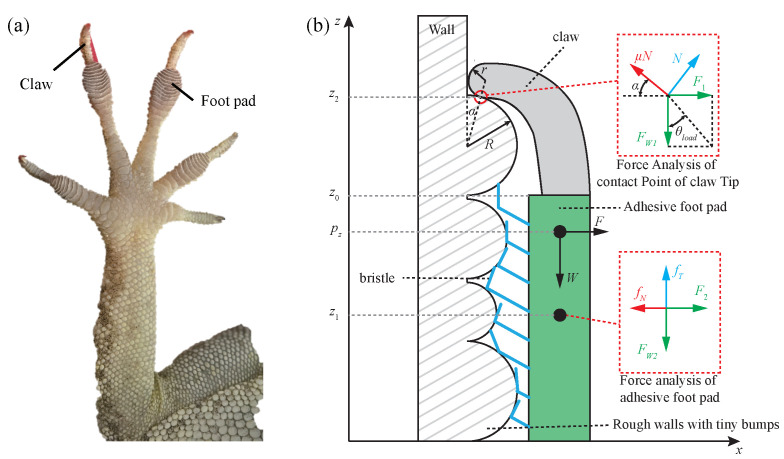
(**a**) Photograph and (**b**) adhesion model of the toe of an anole lizard.

The strong attachment ability may be one of the critical factors for anole lizards to survive in high winds, and it is the guarantee to realize stable climbing movement. Unlike ordinary geckos, the slender toes of anole lizards not only have a toepad composed of a multi-layer adhesive bristle structure to provide sufficient adhesion, but they also have a strong claw structure to provide grasping force [32], as shown in Figure 1a. Therefore, the adhesion model of the toe is established considering the joint action of claws and adhesive toepads, as shown in Figure 1b.

The wall’s convex peak and the claw’s tip are approximately spherical surfaces [33,34,35], and the radius of the convex peak and the tip of the claw at the contact point are *R* and *r*, respectively. The moment of $\tau (p_{z})$ around the toe can be calculated as follows:

(1) $$\tau \left(p_{z}\right)=\left(z_{2}-p_{z}\right)\left(\mu N\cos\alpha -N\sin\alpha -F_{1}\right)-\left(z_{1}-p_{z}\right)\left(f_{N}-F_{2}\right)$$

where $p_{z}$ is a certain point of the toe, $z_{1}$ and $z_{2}$ are the force center point of the toepad and the contact point between the claw tip and the wall, respectively, $F_{1}$ and $F_{W2}$ are the components of the external overturning force *F* on the claw tip and the adhesive toepad, respectively, with $W=F_{1}+F_{2}$, $f_{N}$ is the adhesion force of the toepad perpendicular to the wall, *N* is the supporting force of the convex peak on the wall to the claw tip, μ is the friction coefficient between the claw tip and the convex peak, and α is the contact angle that is between the tangent direction of the contact point of the claw tip and the convex peak and the vertical wall direction.

Consider the action point where the torque is equal to zero, that is, $\tau (p_{z})=0$, and hence, $p_{z}$ can be obtained as follows:

(2) $$p_{z}=\frac{z_{1}\left(f_{N}-F_{2}\right)-z_{2}\left(\mu N\cos\alpha -N\sin\alpha -F_{1}\right)}{\left(f_{N}-F_{2}\right)-\left(\mu N\cos\alpha -N\sin\alpha -F_{1}\right)}$$

**Figure 2:**
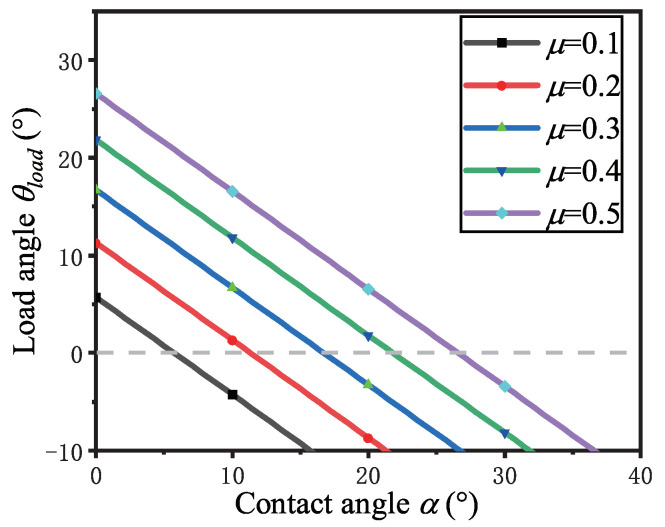
Relationship between the contact angle, the load angle, and the friction coefficient.

The following equations can be obtained considering mechanical equilibrium conditions:

(3) $$\left\{\begin{array}{c} F_{1}=\mu N\cos\alpha -N\sin\alpha \\ F_{W1}=\mu N\sin\alpha +N\cos\alpha \\ f_{T}=F_{W2}\\ f_{N}=F_{2} \end{array}\right.$$

where $F_{W1}$ and $F_{W2}$ are the component forces of the gravity component *W* on the claw tip and the adhesive toepad, respectively, and $W=F_{W1}+F_{W2}$. By solving Equation (3), the following equation can be obtained:

(4) $$\tan\theta_{load}=\frac{F_{1}}{F_{W1}}=\frac{\mu \cos\alpha -\sin\alpha }{\mu \sin\alpha +\cos\alpha }=\frac{\mu -\tan\alpha }{\mu \tan\alpha +1}$$

where $\theta_{load}$ is the load angle of the claw tip, $f_{T}$ is the tangential adhesion force of the toepad along the wall, and $\tan\alpha =\frac{r}{\sqrt{r^{2}+{\left(r+R\right)}^{2}}}$. The relationships between the contact angle, the load angle, and the friction coefficient are shown in Figure 2.

When the load angle $\theta_{load}=0$, the friction coefficient can be deduced from Equation (4) as follows:

(5) μ=tanα

In this case, $F_{1}=0$, and the external overturning force component is entirely offset by the adhesion force produced by the toepad, that is, $F=F_{2}$. In order to remain at rest, the gravity component *W* can be entirely offset by the grasping force generated by the claw within the stiffness range of the claw.

When μ<tanα, the component of the supporting force in the outward direction of the vertical wall is more significant than that in the inward direction of the vertical wall provided by friction; that is, μNcosα−Nsinα<0, and the part of the claw will tend to overturn. In order to maintain balance, the toe cushion must have sufficient adhesion to withstand the external overturning force and the torque change caused by the additional supporting force component,

(6) $$\tau \left(p_{z}\right)=-\left(z_{2}-p_{z}\right)\left(\mu N\cos\alpha -N\sin\alpha \right)-\left(z_{1}-p_{z}\right)f_{N}$$

At this point, the zero moment point $p_{z}$ position is:

(7) $$p_{z}=\frac{z_{1}f_{N}+z_{2}\left(\mu N\cos\alpha -N\sin\alpha \right)}{f_{N}+\left(\mu N\cos\alpha -N\sin\alpha \right)}$$

The zero moment point and $z_{1}$ point are close to or near the contact boundary $z_{2}$. It is an additional burden on the adhesion of the toepad to maintain contact with the claws. In the case of sufficient adhesion force, the contact of the claws can be released so that the normal adhesion force and shear force generated by the toepad bears the external overturning force component and gravity component, which is more conducive to maintaining balance.

When μ>tanα, μNcosα−Nsinα>0 can be obtained. To maintain mechanical balance, the external overturning force component *F* is balanced with the typical balance, and the load angle $\theta_{load}$ is more significant than zero. Therefore, in the case of a specific adhesive force provided by the toepad, this situation will provide a more remarkable ability to resist external overturning forces so that the lizard can maintain stable adhesion under the interference of strong winds.

Based on Equations (4), (5), (7) and Figure 2, the following conclusions can be drawn:

(1) When the contact angle α is constant, the load angle $\theta_{load}$ increases with the increase of friction coefficient μ, and:
  (a) When μ<tanα, the load angle $\theta_{load}<0$; the claw structure part can not maintain stable adhesion. If it bears the gravity component *W*, it will produce an additional normal overturning force, offsetting the zero moment point to the edge of the contact range or beyond the contact boundary, resulting in toe attachment instability.
  (b) When μ=tanα, the load angle $\theta_{load}=0$; the claw bears all the gravity components *W*, the external overturning force component is entirely borne by the toepad, the zero moment point is kept inside the contact boundary, and the toes are stably attached.
  (c) When μ>tanα, the load angle $\theta_{load}>0$; the claw can bear all the gravity components *W* and share part of the normal overturning force. When the toepad adhesion force is specific, the toes can more remarkably resist the external overturning force and keep the toes attached stably.
(2) When the friction coefficient μ is constant, the load angle α increases with the decrease of the contact angle $\theta_{load}>0$; that is, when the wall roughness is constant, the smaller the claw tip is, the more stably the toe can maintain adhesion.

When the load angle $\theta_{load}>0$ is constant, the contact angle α increases gradually with the increase of friction coefficient μ; that is, the proper increase of friction coefficient is beneficial for maintaining stable adhesion of toes.

### 2.2. Modeling the Desorption Mechanics of Anole Lizard Toes

**Figure 3:**
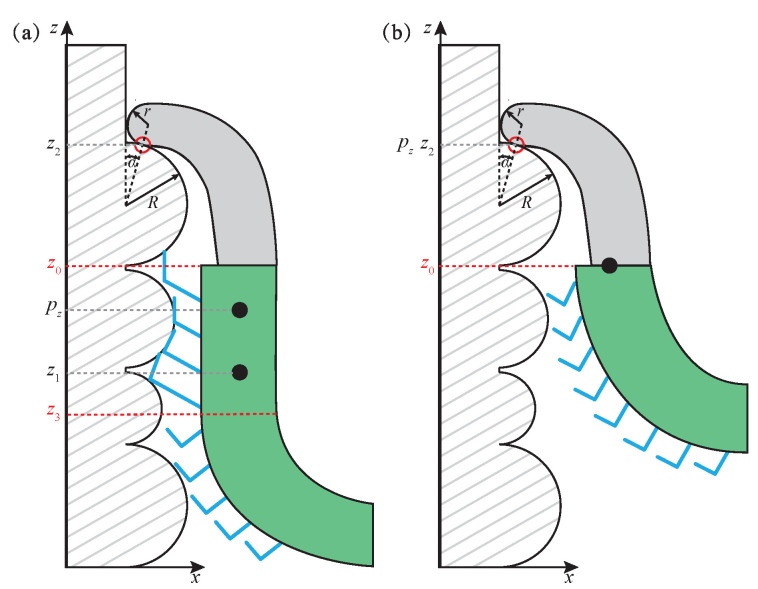
Toe desorption model of anole lizard, including (**a**) incomplete desorption state and (**b**) complete desorption state.

The passive curling of toes during climbing is similar to peeling off the wall with Kendall adhesive [36]. The toepad is composed of many tiny bristles, which have the same structure and anisotropy as the prominent gecko toe bristles, so it is desorbed in the form of detaching. For a vertical wall, the dynamic model of the desorption process of anole lizard toes is shown in Figure 3, where $z_{0}$ to $z_{3}$ are the toepad adhesion points.

The stable attachment state of the toe is measured by whether the position of the zero moment point $p_{z}$ is kept in the toe contact area. As mentioned earlier, in the attachment area, the zero moment point is the same as that of Equation (2).

When μ=tanα, the toe attachment state is shown in Figure 3a; the toepad adhesion area decreases gradually in the process of toe rolling. Since the claw always bears the gravity component, the supporting force *N* remains the same, and the contact angle remains the same without position change; μNcosα−Nsinα=0, $F_{1}=0$, $F_{2}=F$, and the following equation is obtained:

(8) $$\tau \left(p_{z}\right)=\left(z_{1}-p_{z}\right)\left(f_{N}-F\right)$$

In the initial stage of toe rolling, the reduction of adhesion area is slight, and the adhesion force is sufficient to bear the external overturning force component, namely $F_{N}=F$; then $\tau (p_{z})=0$, and zero torque point $\tau (p_{z})$ can exist anywhere in the adhesion area from $z_{0}$ to $z_{3}$. In the later stage of toe rolling, the adhesion area decreases significantly, and when $F_{N}<F$, the toe falls off as a whole and loses its stability.

When μ<tanα, release of the claw is more in line with stability requirements because contact of the claw brings an additional burden. At this time, the gravity component and the external overturning force component are all borne by the toepad, and the magnitude of the external overturning force and the gravity component affect the toe attachment state. When the toe rolls so that the toepad cannot bear gravity or external overturning force, any one of the toes will suddenly lose stability and fall off as a whole.

When μ>tanα, the zero moment point $p_{z}$ position can be obtained as follows:

(9) $$p_{z}=\frac{z_{1}\left(f_{N}-F_{2}\right)-z_{2}\left(a-F_{1}\right)}{\left(f_{N}-F_{2}\right)-\left(a-F_{1}\right)}$$

where a=μNcosα−Nsinα is constant. In the initial stage of toe roll and desorption, the position of $z_{2}$ remains unchanged, as shown in Equation (9); with the approach of $z_{3}$ to $z_{2}$, $z_{1}$ and $p_{z}$ gradually approach $z_{2}$; if a≥F, the toepad will eventually fall off entirely, and the gravity component *W* and the external overturning force component *f* will be borne entirely by the claw, as shown in Figure 3b. If a<F, the toe will be detached as a whole before the adhesive pad falls off ultimately, and the stable range is more significant than that of μ=tanα.

Therefore, it can be concluded that the friction coefficient is an essential factor in determining the stable range of foot adhesion and desorption, and the rolling process of flexible adhesive toes ensures the continued stability of the desorption movement. The cooperation between claws and adhesive toepad improves the resistance of an anole lizard to external disturbance.

### 2.3. Wind Resistance Mechanism of Anole Lizard

When a lizard is in a strong wind, it instinctively points its head in the direction of the wind to keep its balance. This makes less of the lizard’s body face the wind, which reduces wind resistance. At this time, if there is no obstacle in front of the lizard, under continuous strong wind, the effect of airflow is the same on both sides of the lizard’s body because the two sides of the body are symmetrical, but the abdomen and back of the lizard have different physical structures. Therefore, it is speculated that the difference between the abdomen and back has a specific impact on body stress [37,38].

**Figure 4:**
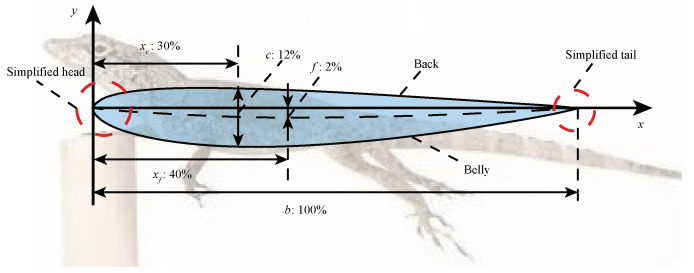
Equivalent cross-section model of anole lizard body.

According to the statistical data of Donihue et al. [31] on the body sizes of lizards that survived the hurricane, an equivalent plane model of the cross-section in the lateral direction is drawn, as shown in Figure 4. The body proportion relationship is developed by simplifying the head and tail and removing the limbs: b=100 mm is the length of the anole lizard body, c=12 mm and f=2 mm represent the thickness of the belly and flexure, respectively, and their ratio to the length of the anole lizard body represent the relative thickness $\frac{c}{b}=12\%$ and the relative flexure $\frac{f}{b}=12\%$, respectively. Thus, $x_{c}$ and $x_{f}$ represent the lengths of point *c* and point *f* from the origin, respectively. It can be seen that the section of the adjusted body shows a wing shape, which is influenced by the wind speed during the windward process and is subject to pressure from the back to the abdomen.

**Figure 5:**
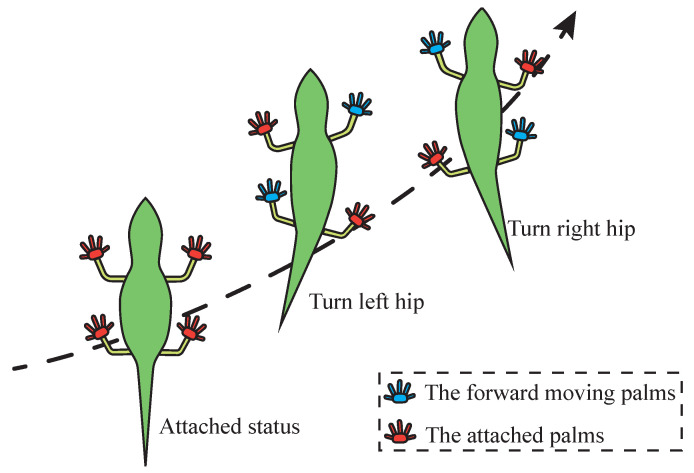
Forward movement strategy of an anole lizard.

The forward movement of an anole lizard is different from that of ordinary quadruped reptiles. Its forward movement does not simply rely on alternating movement of its limbs; rather, the movement is characterized by the lizard’s excellent spine flexibility. In forward movement, rotation of the spine drives the hip to rotate. As shown in Figure 5, when the hip turns left, the left hind limb is driven forward, and the right forelimb extends forward, completing a step forward. After that, the soles of the feet are attached and stabilized, and then the right hip is turned to complete the forward movement of the right hindlimb and the left forelimb, at which time a movement cycle ends alternately.

The spine of an anole lizard is a flexible structure with continuous bending deformation formed by a multi-segmented bone structure, which can effectively slow down its vibration in the process of force transmission during movement. It is assumed that passive bending of the spine in an upwind environment can slow down the body vibration caused by the wind and ensure stability during climbing. At the same time, the tail of an anole lizard has variable curvature, which plays a role in adjusting balance during movement. Therefore, it is assumed that the change in the tail curvature results in a force change in the body. This may play a role in reducing the influence of wind on the body and enhancing the lizard’s adhesion ability.

## 3. Design of the Anole Lizard-Inspired Climbing Robot

**Figure 6:**
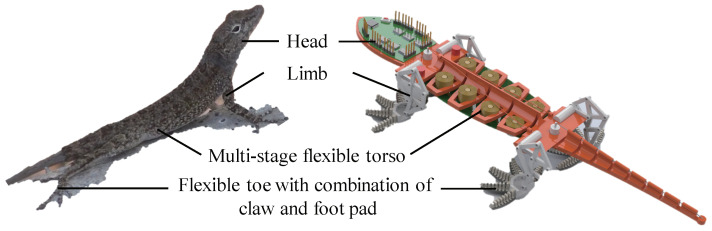
Design diagram of robot that imitates an anole lizard.

To improve the climbing stability of a climbing robot in high wind, an anole lizard-like climbing robot with a flexible toe, a bionic limb, and a multi-stage flexible torso is designed based on the shape and movement of anole lizards, as shown in Figure 6.

**Figure 7:**
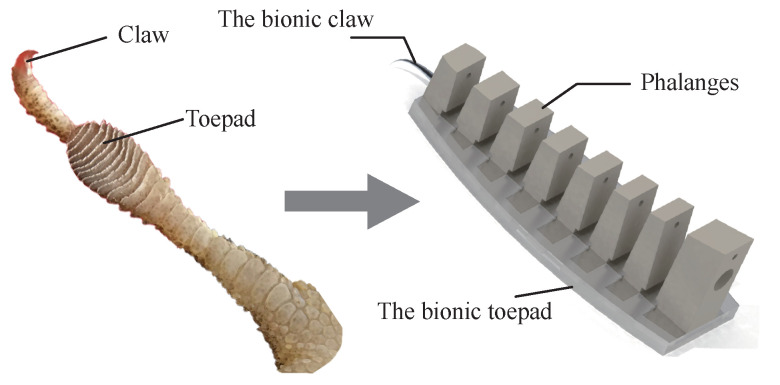
Simplified model of the toe.

Based on the the continuous deformation ability of anole lizard toes, three long phalanx bones are evolved into phalanx bones with continuous changes in height, and traction lines are passed through the middle of each phalanx bone to imitate muscle. The toe is bent by tightening and releasing the traction wire. Based on the directional adhesion characteristics of the flexible toepads, anisotropic flexible adhesive materials are prepared to cover the phalanges. It should be noted that this material has good adhesion properties on a specific surface, i.e., it has poor surface adaptation, resulting in relatively-weak climbing performance of the robot on other surfaces. Imitating the climbing movement mode of the claw and toepad adhesion, a fish hook is used as the claw structure embedded in the front of the toe bone, as shown in Figure 7.

**Figure 8:**
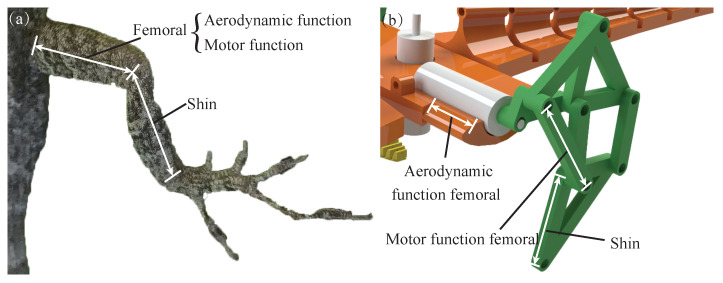
(**a**) Photograph and (**b**) simplified limb model of the anole leg.

The legs of an anole lizard have three main joints: ankle, knee, and hip. For the robot, the leg does not need large-scale movement; it only needs to meet the motion trajectory of the foot on the plane. Therefore, the ankle joint and knee joint of an anole lizard can be replaced by passive rotation pairs, and the hip joint can be simplified to be driven by only a single motor, which can reduce the number of motors and reduce the quality of the whole machine. The bionic leg has the agile characteristics of dexterity, high speed, lightness, and high efficiency, and its most significant characteristic is less freedom, especially single-degree-of-freedom drive. However, the mechanism that imitates the upright bionic leg through the connecting rod already contains the motion function of the femur and tibia. The leg mechanism of the lizard has a specific aerodynamic function in high wind, so the motion function of the femur can be separated from the aerodynamic function, and the moving mechanism can be simplified, as shown in Figure 8. In addition, the motion track of the designed leg structure is D-shaped, which is convenient for the desorption and adhesion of the toes.

Through analysis of the structure of the anole lizard, it is found that the radian of the abdomen has an important influence on aerodynamic characteristics. In confronting the airflow, the bulging of the abdomen provides the lizard with pressure pointing to the wall, enhancing toe adhesion. Therefore, the design of abdominal modeling plays an important role. The abdomen is thus made of flexible, deformable, magnetically sensitive rubber.

**Figure 9:**
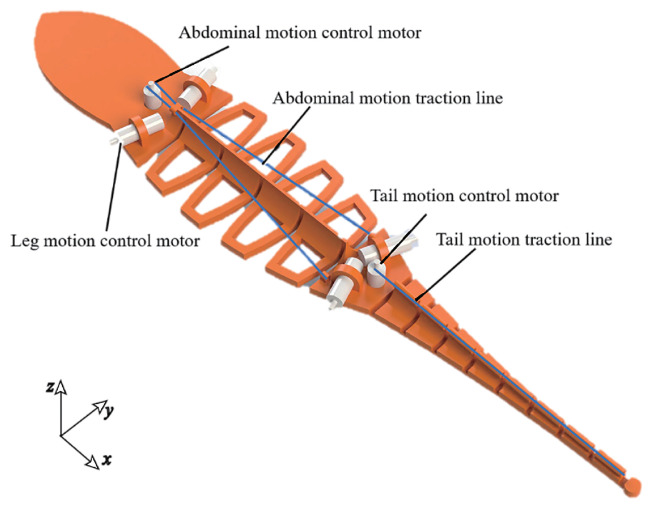
Simplified model of the torso.

The swing of the spine plays a vital role in the stable climbing behavior of the lizard. However, the rigid connection of the rotating joint is not conducive to the robot imitating the lizard to fully use its body structure to passively adjust its shape to change the aerodynamic characteristics of the body. In strong winds, anole lizards relax their entire body to fully use the energy generated by the oscillation of the airflow behind their perch to reduce the load required for foot attachment. Therefore, the flexible rotation mechanism of the spine is the key to the design of the body mechanism. As shown in Figure 9, the body mechanism of the anole lizard climbing robot includes a spine and ribs that rotate around the Z axis, a motor installation position controlled by the motion of the limbs, the abdomen, a head with a pneumatic structure, and a tail rotating around the Y axis. The rotation of the spine drives the abdominal traction wire to deflect through the rotation of the motor, while the tail is bent and deformed by driving the tail motion traction wire through the single motor of the tail; thus, the aerodynamic characteristics of the body change.

**Figure 10:**
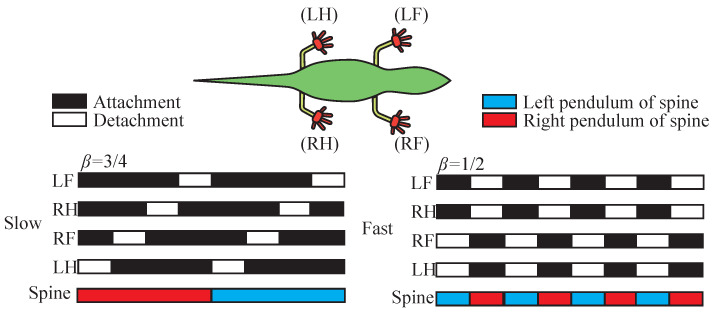
Motion–gait phase relationship of the anole lizard-inspired climbing robot; β is the ratio of adhesion time to the sum of adhesion and detachment time.

The degree of freedom of this climbing robot is reduced by a novel design of the limbs, and there are only six joints to be controlled: four limb joints, one spine joint, and one tail joint. In addition, control motors with high torque and light self-weight are employed as the driving module for the climbing system. The motion–gait phase relationship of the anole lizard-inspired climbing robot is shown in Figure 10.

## 4. Simulation Analysis and Experimental Verification

### 4.1. Simulation of the Wind Resistance Mechanism of Anole Lizard

**Figure 11:**
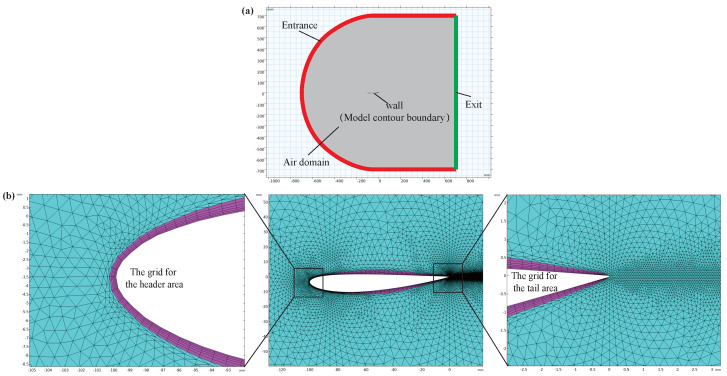
Simulation parameter setup, including (**a**) air domain setting and (**b**) mesh generation of the section abstract model.

In order to reveal the influence of the particular shape and structure of the anole lizard on its wind resistance performance under high-speed airflow, the dynamic behavior of its body in airflow is simulated by COMSOL multiphysics field simulation software. The section length *b* is set to 100 mm at the position of the body length $x_{f}=40$ mm, and the distance between the middle arc (the midpoint of the connection between the upper and lower surfaces) and the body length reaches a maximum value of 2 mm at the position of body length $x_{c}=30$ mm. The air domain is set as shown in Figure 11a. In order to ensure uniform airflow, the air domain should be large enough; thus, the left semicircle with a radius of 700 mm is designated, the upper and lower boundary of a rectangle with a width of 700 mm are set, and the height of the rectangle is the right boundary of 1400 mm as the exit. As shown in Figure 11b, the simulation model meshed. In order to obtain better accuracy, a quadrilateral mesh is used to deal with the contour boundary of the model, and the triangular mesh is used in the rest of the air domain to reduce calculations and ensure calculation accuracy.

The air domain is set as an ideal gas with a temperature of 20 °C, the mouth wind speed is 50 m/s, and the outlet pressure gauge pressure is 0 (that is, the pressure is equal to the atmospheric pressure). To solve the model, a better initial value is obtained by using the k−ϵ turbulence model, which has a reasonable convergence rate and relatively low memory requirements; then, the SST model is used to solve for accurate results near the wall, and the turbulence intensity is set to medium.

Perch obstacles can be simplified to the classic case of flow around a cylinder, but in order to directly show the flow-field environment of an anole lizard, the Fluent module in the Ansys student version is used to simulate and analyze the flow around the back of the perch. In order to form a comparison, the simulation process should be consistent with the experimental conditions of Donihue et al. [31]. Using the SST model, ideal air fills the air domain at 20 °C; the properties of the gas are shown in Table 1.

**Figure 12:**
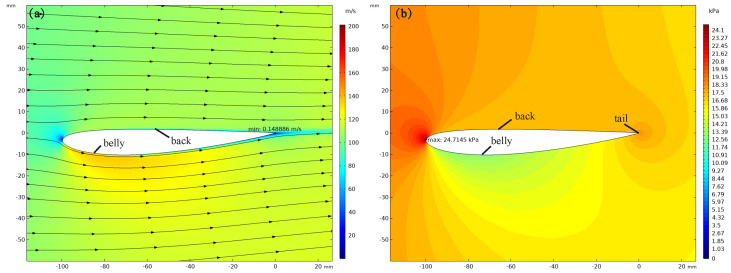
(**a**) Velocity distribution and (**b**) pressure distribution around the simplified model.

Figure 12a shows the velocity distribution around the simplified model. The results show that the airflow near the model is accelerated. The maximum air velocity around the model appears below the abdomen. In contrast, the minimum velocity appears in the tail, the average velocity of the abdomen is between 120–140 m/s, the back velocity is between 80–110 m/s, and the average air velocity of the back is lower than that of the abdomen; as shown in Figure 12b, when high air flows through the body of an anole lizard, the average pressure on the back is more significant than the average pressure on the abdomen. The pressures on the abdomen and back are in the range between 10.91–13.39 kPa and 16.7–17.5 kPa, respectively. Thus, the model will be subjected to pressure on the back pointing to the abdomen.

According to the Bernoulli principle, the change of air velocity is bound to change air pressure around the simplified model, which can be expressed as follows:

(10) $$p+\frac{1}{2}\rho v^{2}+\rho gh=C$$

where *p* is the pressure of a certain point in the fluid, *v* is the fluid velocity at that point, ρ is the fluid density, *g* is the gravitational acceleration, *h* is the height of the point, and *C* is a constant that represents the total energy of the airflow. As can be seen from Equation (10), when the air density and height are constants, the velocity around the abdomen is large, so the pressure near the abdomen is less than the distant atmospheric pressure. In addition, the pressure on the back will be greater than the pressure on the abdomen, resulting in a positive pressure pointing from the back to the abdomen. The classical friction law points out that when the friction coefficient is constant, the positive pressure is positively correlated with the magnitude of friction. Therefore, the increase of positive pressure will produce more friction to resist the positive pressure caused by the airflow on the body of the lizard, and when the resultant of friction, foot adhesion, and grasping force is greater than the pressure, the lizard can achieve stable climbing; namely,

(11) $$\mu F_{N}+F_{a}+F_{c}\geq F_{T}$$

where $F_{N}$ is the positive pressure from the back to the abdomen, μ is the friction coefficient, $F_{a}$ is the tangential adhesion force of the foot, $F_{c}$ is the tangential grasping force produced by the toes of the lizard, and $F_{T}$ is the pressure caused by the airflow on the front of the lizard.

One of the possible reasons for an anole lizard surviving in strong wind is that when high wind flows through its body, the air velocity around the body changes because of its particular body shape. A pressure difference is formed between the back and abdomen; hence, the friction force and tangential adhesion force of the feet of the anole lizard are improved, and stable attachment of the anole lizard is ensured.

**Figure 13:**
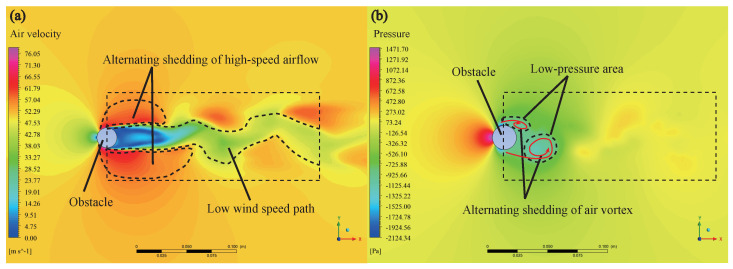
Air velocity and pressure behind the obstacle. (**a**) the airflow behind the obstacle has relatively low velocity and sheds alternately from both sides after encountering the obstacle, forming a classical Karman vortex street; (**b**) the alternate shedding of air vortices creates alternating high and low-pressure paths with specific wavelengths and frequencies.

As shown in Figure 13a, the air velocity behind an obstacle is relatively low with a wind speed of 50 m/s. The air flow alternately falls off from both sides after encountering the obstacle, forming the classic Karman vortex street, creating a fluctuating low wind speed path behind the obstacle. The lower air velocity behind the obstacle means that the air resistance to the lizard will be significantly reduced, which provides a natural shelter for the lizard. However, the wind speed is still in the range of 0 m/s to 30 m/s when the lizard length is less than the obstacle size. If there is a confrontation with the lizard, the lizard will still produce excellent resistance. The periodic fluctuation of the wind speed behind the obstacle will also lead to periodic fluctuation of air pressure in the region, as shown in Figure 13b. Therefore, the addition of obstacles affects the uniform flow of air, and the alternating shedding of air vortices forms a path of alternating high and low pressure with specific wavelengths and frequencies.

**Figure 14:**
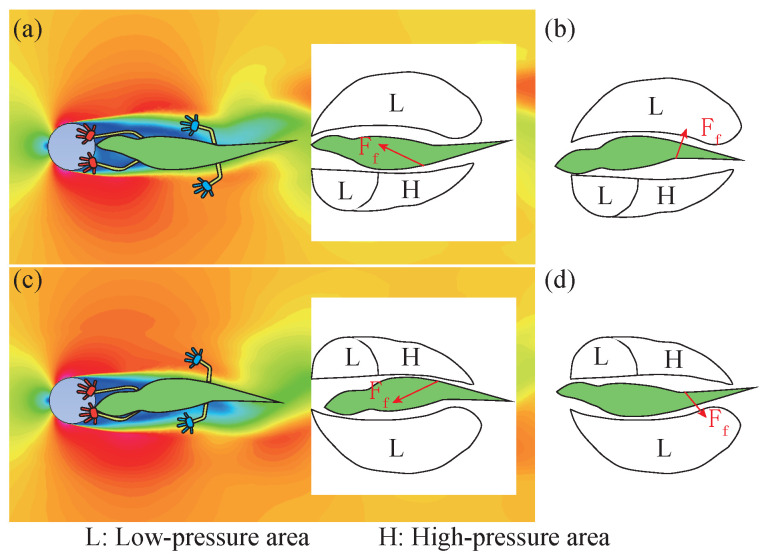
Influence of air pressure on a lizard behind an obstacle. When the pressure on the left is higher than that on the right, (**a**) the lizard swings to the left, (**b**) the lizard swings to the right; when the pressure on the right is higher than that on the left, (**c**) the lizard swings to the right, and (**d**) the lizard swings to the left.

The periodic fluctuation formed by alternating shedding of the air vortex behind the obstacle is bound to affect the anole lizard behind the obstacle and cause periodic lateral force on its body. If no measures are taken, the wind will harm its climbing stability. In a video shot by Colin M. Donihue of a wind experiment on an anole lizard, when the wind speed is high, the anole lizard uses its forelimbs to hold the obstacle, the hindlimbs are released, and the whole body is relaxed to meet the high wind, producing the movement posture of swinging left and right in the wind, and maintaining attachment under high wind. Several conjectures violate the conventional conjecture of most people: first, the anole lizard releases its hind limbs in high wind, reducing the attachment of the soles of its feet with strong adhesion from four to two, weakening its adhesion ability; and second, the body is in a state of relaxation, rather than struggling to achieve a tight grip. Because of its particular movement gait, an anole is placed behind the obstacle, and its stress state is discussed. Figure 14 shows an analysis of its left and right pendulum states.

When the air vortex is alternately shed, a pressure difference is formed on the left and right sides that acts directly on the body of the anole lizard, producing a force in the rightward direction. If the anole lizard swings to the left, a force forward to the right is generated due to the tail pinch, as shown in Figure 14a. As shown in Figure 14b, when the lizard swings to the right, the angle between the tail causes it to produce a backward-to-right force. When the pressure on the right is higher than the pressure on the left when the lizard swings its tail in the direction of high pressure, a forward-to-left force will be generated, as shown in Figure 14c, and a backward-to-left force will be generated when the tail is swung to the right, as shown in Figure 14d.

In general, when the anole lizard swings in the direction of high pressure, the force on the whole body will be subjected to a forward component, and generation of the forward force will counteract the resistance caused by the positive flow to a certain extent. If an active swing accompanies the anole lizard’s body at the same frequency as the air vortex, the forward component force can be even greater than the resistance of the forward flow, making it move forward like a fish swimming in the water so that the soles of the feet need little adhesion.

### 4.2. Experimental Verification

**Figure 15:**
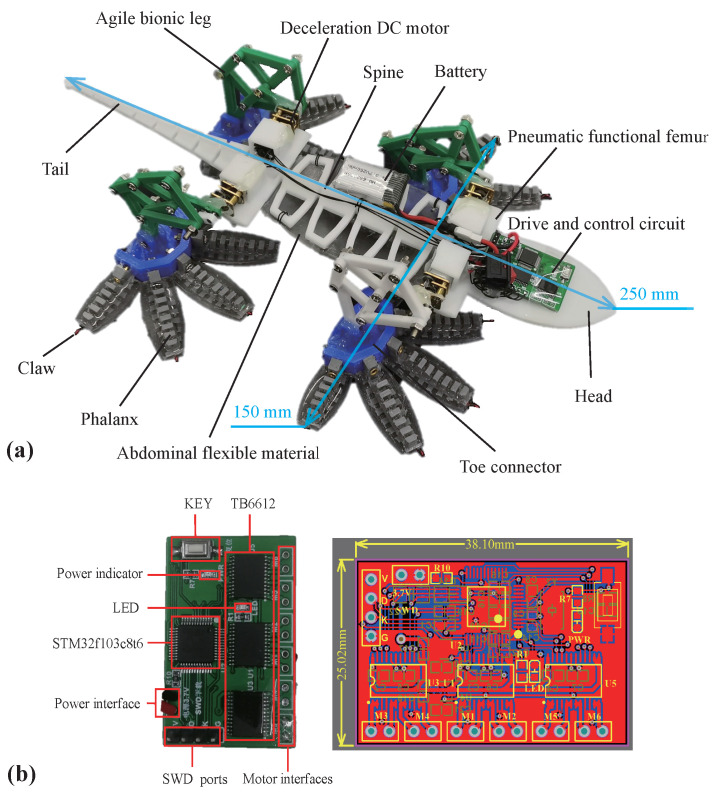
(**a**) Photograph of the anole lizard-like climbing robot and (**b**) drive and control circuit.

Based on the design in Section 3, we made an anole lizard climbing robot with multi-stage flexible mechanisms such as toe bones, spine, coccyx, as well as flexible paws of the abdomen, claws, and toepads with the ability of deformation adjustment, agile bionic legs, and body load mechanism, with a total length of 250 mm, a width of 150 mm, and a weight of 0.235 kg. The physical diagram is shown in Figure 15a. The climbing robot is driven by four deceleration DC motors (TeleSky GA12-N20, Haoyun Technologies Co., Ltd., Guangzhou, China) with high torque and small size, whose control and drive circuits are shown in Figure 15b. An STM32F103C8T6 is used as the control core to drive the four motors, and a TB6612 chip is used to control the speed and rotation time of the four motors through PWM to output regular gait control signals.

**Figure 16:**
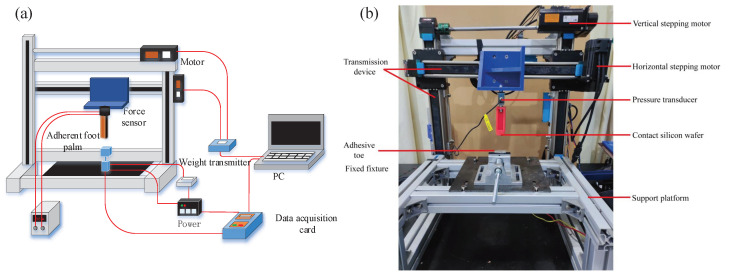
Test platform for the adhesion ability of the foot: (**a**) schematic diagram and (**b**) experimental setup.

First of all, we test the foot adhesion ability of the robot, and the hardware part of the test system is mainly composed of motion- and data-sampling systems. The schematic and physical diagram of the test platform are shown in Figure 16 and are mainly composed of a motion system and a data sampling system. The motion system consists of two stepper motors, a guide rail, and a controller, and the data acquisition system consists of a high-precision S-type force sensor (resistive strain sensor), a weight transmitter, a data acquisition card, and computer software. The two linear motors generate linear motion in two orthogonal axes, i.e., parallel and vertical to the ground, namely, the horizontal and vertical axes, or X- and Y-axes, respectively. The force sensor is installed on the linear motor platform along the Y-axis, and the adhesion force is obtained by measuring the contact force between the material and the rigid plane in real-time.

**Figure 17:**
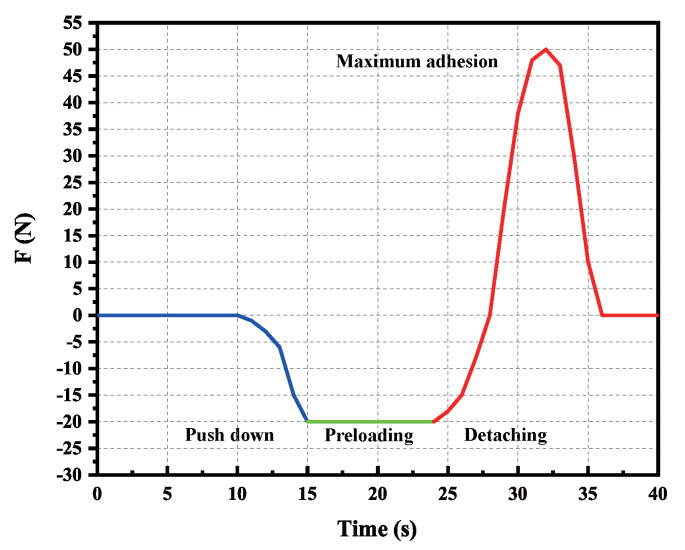
Experimental results of toe adhesion.

The test procedure is designed as follows: First, the test sample is fixed to the top of the bracket, and then, the vertical motor is driven to move the connecting rod downward at a constant speed and stop when the contact force reaches −20 N. Second, the vertical motor is driven to make the connecting rod move upward at a constant speed after pressing for a period of time. Finally, the contact force is acquired using the force sensor and recorded with data acquisition software. The experiment is repeated 5 times, and the average value of the result is used. The experimental results are shown in Figure 17. The adhesion experiment shows that the toe has a specific ability, and the maximum adhesion force appears near 50 N under the pre-press pressure of 20 N, which meets the adhesion performance requirements.

**Figure 18:**
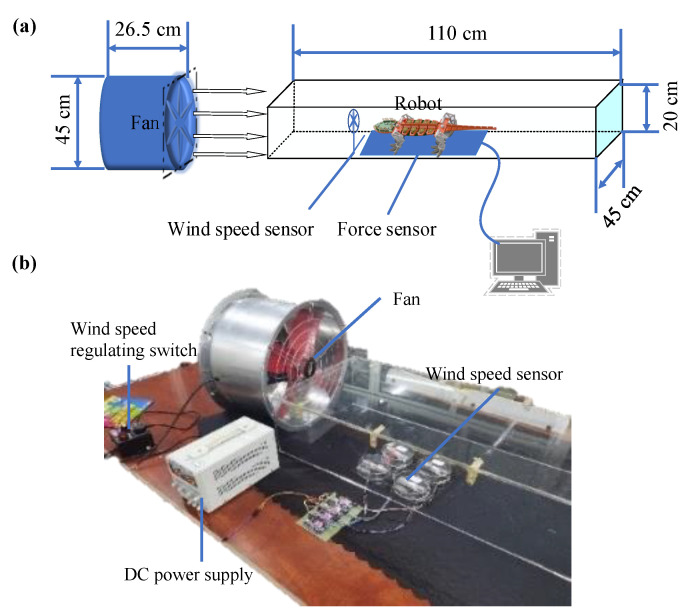
Small wind tunnel test platform: (**a**) schematic diagram and (**b**) experimental setup.

Wind resistance performance is tested by building a small wind tunnel experimental platform. The schematic and physical diagram of the test platform are shown in Figure 18. The test platform mainly comprises airflow generation, a rectifier system, and a data sampling system. The airflow generation system includes a fan and an AC fan governor whose wind speed can reach 15 m/s. The rectifier system comprises multiple suckers to ensure the wind can reach a local uniform speed after the flow. The data acquisition system includes a wind-speed sensor (FM-YLF, 0–20 m/s, Shenzhen Youxin Electronic Technology Co., Ltd., Shenzhen, China), a force sensor (BT-130, 0–10 N, Shenzhen Youxin Electronic Technology Co., Ltd., China), and computer software. During the test, the robot is placed above the force sensor, the wind speed of the fan is slowly adjusted, and the data returned by the wind speed sensor and the force sensor are recorded by the computer software.

**Figure 19:**
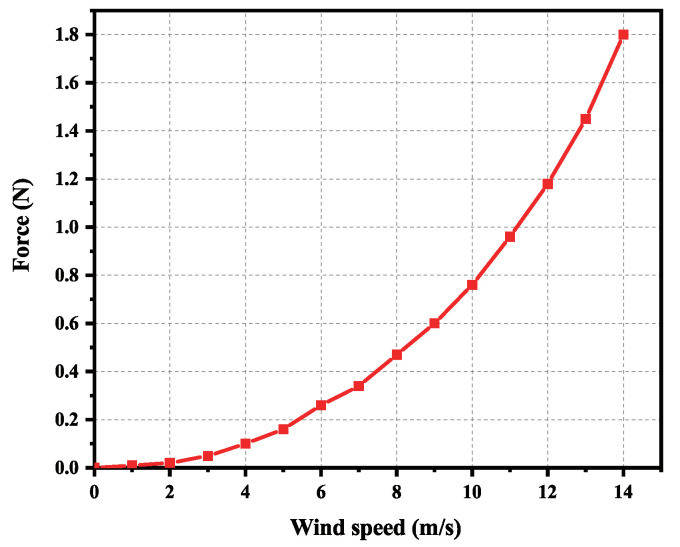
Robot barrier-free upwind test data.

The anole lizard-imitating climbing robot is placed on the experimental platform, and the data change can be easily observed by setting the platform to 0 value through the peeling button. The wind speed can be set to 0–14 m/s. As the wind speed increases, the pressure on the robot pointing to the wall also gradually increases. When the wind speed is 14 m/s, the pressure can reach 1.8 N, 76.6% of its weight. The barrier-free upwind test data of the robot are shown in Figure 19. The experimental results show that the anole lizard climbing robot has stable attachment ability in high winds, and the greater the wind speed is, the greater the attachment capacity is.

## 5. Conclusions and Future Works

This study focused on proposing an anole lizard-like climbing robot inspired by its superior wind resistance. The adhesion and desorption models of the anole lizard’s toe are first proposed, which demonstrated that the friction coefficient is the critical factor in determining the stability of the toe adhesion and desorption, and the cooperation of claw and adhesive toepad improves the ability to resist high wind. In addition, an anole lizard-like climbing robot with soft toes, limbs, and a multi-stage flexible torso was designed based on the simplified body model.

Then, the aerodynamic behaviors of the climbing robot in high-speed airflow were studied using COMSOL and ANSYS finite element analysis methods. The results demonstrated that when there is no obstacle, the robot generates the normal force by adjusting the abdomen’s shape to create an air pressure difference between the back and the abdomen. When there is an obstacle, a component force to resist the frontal wind is obtained through periodic oscillation of the spine and tail resulting from the vortex paths generated by the airflow behind the obstacle. Furthermore, the results confirm the hypothesis that the anole lizard improves its adhesion and stability through abdominal adjustment and trunk swing.

Finally, an anole lizard-like climbing robot prototype with a length of 250 mm, a width of 150 mm, and a weight of 0.235 kg was fabricated. In order to verify its adhesion and wind resistance performances, an adhesion and desorption test platform and a small wind tunnel test system were developed. The test results show that the adhesion force is equal to 50 N when the pre-pressure is 20 N, and the normal pressure of the proposed robot can reach 76.6% of its weight in a high wind of 14 m/s.

Compared with other climbing robots, our proposed robot has a flexible abdominal structure, which allows the robot to work stably in strong winds. However, due to the poor surface adaptability of the adhesion material used for the toepads, the climbing performance of this robot is relatively weak on walls with different roughness and inclination angles. Therefore, we will further improve the robot’s attachment method and mechanical structure in future work to ensure its excellent climbing ability on surfaces with different roughness and inclination.

## Figures and Tables

**Table 1:** Gas properties of ideal gas filling air domain at 20 °C.

$C_{p}$ (Specific Heat) ((J/kg)·K)	Thermal Conductivity (W/(m·K))	Adhesion (kg/(m·s))	Molecular Weight (kg/kmol)
1006.43	0.0242	$1.7894\times 10^{-5}$	28.966

## Data Availability

Not applicable.
